# A novel assisted technique for difficult 24-hour esophageal pH-impedance probe placement

**DOI:** 10.1055/a-2832-6011

**Published:** 2026-03-26

**Authors:** Rami Reddy Yalaka, Kondal Reddy Mogili, Abhign Chennamadhavuni, Saivivek Reddy Madhuri, Medha Rao Menneni

**Affiliations:** 1 504303 Department of Medical Gastroenterology, Star Hospitals, Hyderabad, India; 2 562425 Mamata Academy of Medical Sciences, Hyderabad, India

Twenty-four-hour pH monitoring involves the transnasal placement of a thin, flexible probe for the evaluation of gastroesophageal reflux disease 1 2 3 . Excessive probe flexibility may lead to buckling and coiling, particularly in patients with altered anatomy like hiatal hernia, esophageal tortuosity, diverticula, or extrinsic compression or post-surgical changes and those with poor cooperation or a pronounced gag reflex. Common facilitating and alternative maneuvers include adequate lubrication, the swallow-and-advance technique, chin-tuck maneuver and head rotation. We demonstrate a simple technique to improve deliverability in difficult insertions.

**Fig. 1:**
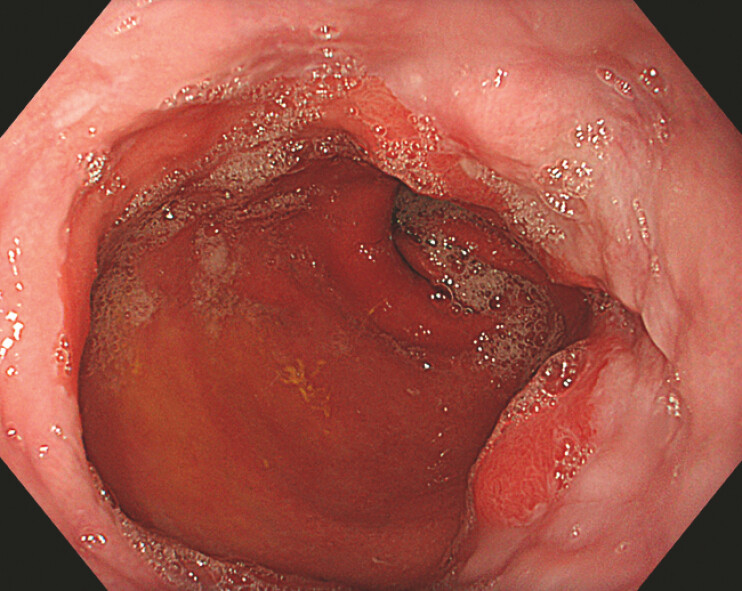
An endoscopic image showing reflux esophagitis and hiatal hernia.

**Fig. 2:**
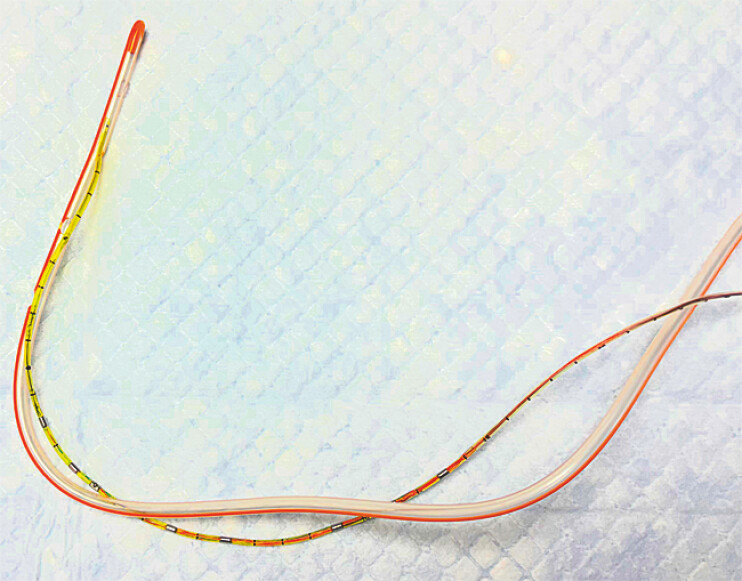
Nasogastric tube-pH probe assembly.

**Fig. 3:**
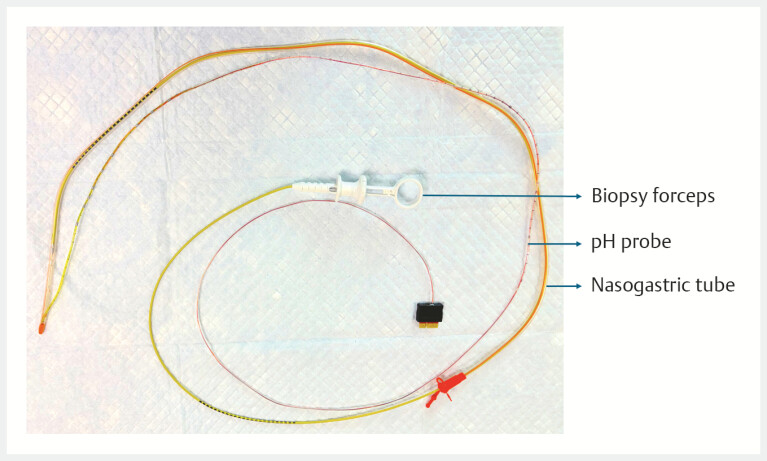
Nasogastric tube-pH probe-biopsy forceps assembly.

**Fig. 4:**
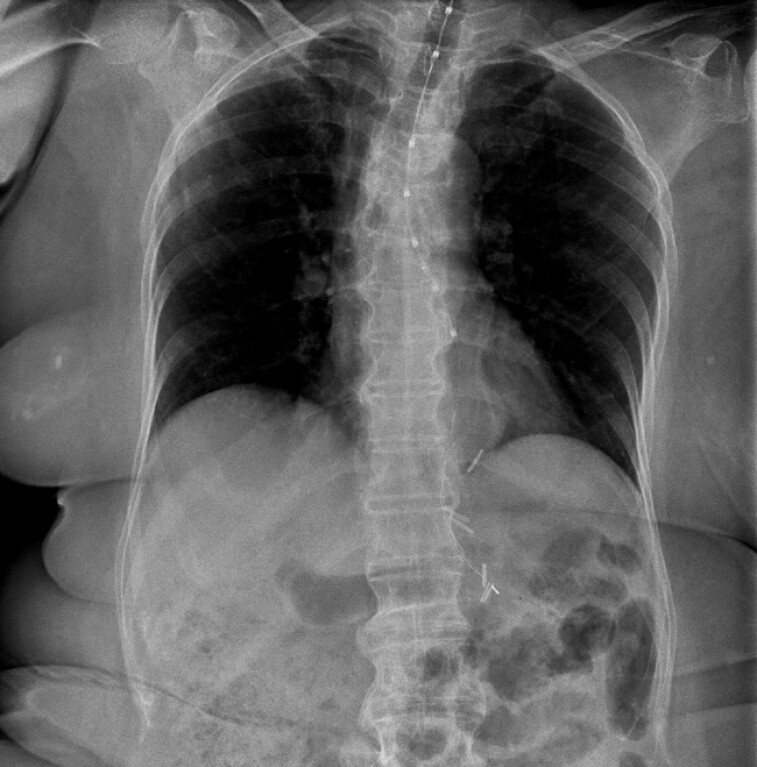
A fluoroscopic image showing the successful pH probe placement (i.e., the esophageal sensor is at +5 cm and the gastric sensor is at –10 cm relative to the EGJ).

A 60-year-old woman diagnosed with reflux esophagitis, sliding hiatal hernia and post-sleeve gastrectomy was referred for 24-hour pH study ( Fig. 1 ). Repeated transnasal attempts using a 6.4-Fr pH probe failed because of persistent coiling at the oropharynx. To facilitate advancement, the distal tip of the pH probe was introduced into the distal side port of a 16-Fr nasogastric tube, creating a temporary nasogastric tube-pH probe assembly ( Fig. 2 ). The nasogastric tube was selected for its relative stiffness and ready availability. The assembly was advanced transnasally under fluoroscopic guidance. As buckling persisted, biopsy forceps were inserted ex-vivo into the nasogastric tube lumen up to 5 cm short of the tip, without exiting the side ports, thereby providing additional stiffness and improved directional control ( Fig. 3 ). This reinforced assembly enabled smooth passage across the esophagogastric junction. Under fluoroscopic guidance, the nasogastric tube was gently advanced while the pH probe was stabilized at the nostril, allowing successful separation ( Video 1 ). The probe was accurately positioned without adverse events ( Fig. 4 ). The patient tolerated the procedure with minimal discomfort, and the study was completed successfully. This simple, reproducible technique may improve procedural efficiency and success in carefully selected patients with difficult pH probe placement.

A novel assisted technique for difficult 24-hour esophageal pH-impedance probe placement.Video 1

Endoscopy_UCTN_Code_CCL_1AB_2AH Endoscopy_UCTN_Code_CCL_1AB_2AC_3AC
